# ﻿*Ophiorrhizareflexa* (Rubiaceae), a new species from a karst region in Guangxi, China

**DOI:** 10.3897/phytokeys.238.116767

**Published:** 2024-02-26

**Authors:** Chao Shang, Jun Xue, Yanjie Yang, Xiaowen Liao, Quanru Liu, Lei Wu

**Affiliations:** 1 College of Forestry, Central South University of Forestry and Technology, Changsha 410004, China Central South University of Forestry and Technology Changsha China; 2 College of Life Sciences, Beijing Normal University, Beijing 100875, Beijing, China Beijing Normal University Beijing China

**Keywords:** China, new taxon, *Ophiorrhiza*, Rubiaceae, taxonomy

## Abstract

*Ophiorrhizareflexa*, a new species from Guangxi, China, is described and illustrated in this study. It is morphologically similar to *O.alatiflora* due to the branched inflorescence, distylous flowers and the tubular-funnelform corolla with five longitudinal wings. The new species can be distinguished from *O.alatiflora* by its erect inflorescence, its smaller and equal-sized calyx lobes 0.5–0.7 mm long, its corolla tubes winged to the middle and the wings straight and its strongly reflexed corolla lobes at anthesis. *Ophiorrhizareflexa* is assessed as least concern (LC) according to IUCN Categories and Criteria.

## ﻿Introduction

*Ophiorrhiza*Linnaeus (1753) is a notably species-rich and taxonomically complicated genus in the family Rubiaceae, comprising about 200–300 species (Deb and Mondal 1997; Chen and Taylor 2011; Li 2020) and mainly distributed in tropical and subtropical Asia (Darwin 1976; Lo 1990; Deb and Mondal 1997; Chen and Taylor 2011; Deng and Huang 2012; Hareesh et al. 2015; Wong 2019; Hu et al. 2021; Schanzer and Nabatov 2022; Liu et al. 2023). Species of the genus are annual or perennial herbs and rarely sub-shrubs that can be easily recognised by their obcordate and compressed fruits, which are dehiscent with two valves along a transverse slit at the top (Darwin 1976; Lo 1990; Chen and Taylor 2011; Wu et al. 2019). Though the genus is well-defined by this distinctive fruit shape, demarcation of species within the genus is sometimes very difficult due to the high morphological variation (Nakamura et al. 2006, 2007; Duan and Lin 2007, 2009; Wu et al. 2017c) and insufficient knowledge of flowers in most species (Hooker 1880; Schanzer 2004; Wu et al. 2017a, b).

China is one of the diversification centres of *Ophiorrhiza*. Approximately 72 species (with 50 endemics) of the genus have been recorded in this country and they are mainly distributed in southern and south-western China, especially in Guangxi Province and Yunnan Province (Chen and Taylor 2011; Huang et al. 2017; Wu et al. 2017a, b, c, 2018; Tu et al. 2018; Duan et al. 2019; Wen et al. 2019; Hu et al. 2021; Liu et al. 2023).

During our field survey in Napo County, western Guangxi, in 2013, we collected a peculiar population of plants in full blossom. The individuals were first identified as *Ophiorrhizaalatiflora* H.S.Lo as they shared similar habitats and morphological characters, such as branched inflorescences, distylous flowers with tubular-funnelform corollas and a corolla with five longitudinal wings. After revisiting the area including the type locality of *O.alatiflora* and further examining the specimens, however, these individuals from Napo County can be distinguished from *O.alatiflora* mainly by their inflorescences which are erect from their earliest developmental stages (vs. drooping when young, then erect), their smaller calyx lobes (0.5–0.7 vs. 0.9–1.8(–2.5) mm long) which are equal in size (vs. usually unequal), the nature of the longitudinal wings on the corolla tube (wings extending from top to middle and straight vs. wings extending along entire length and obviously undulate) and the strongly reflexed corolla lobes (vs. spreading) at anthesis. Therefore, the specimens are assumed to represent an undescribed new taxon, which is here described.

## ﻿Material and methods

Most materials are deposited at the Herbarium of Forest Plants in Central South University of Forestry and Technology (**CSFI**). Herbarium acronyms follow Thiers (2023). Morphological observations of the new species were derived from field observations, as well as a study of dry specimens. The morphological terms employed here follow Chen and Taylor (2011). The conservation status of this new species is evaluated, based on field observations in accordance with IUCN Red List guidelines (IUCN 2023).

## ﻿Taxonomic treatment

### ﻿ Ophiorrhiza reflexa

Taxon classificationPlantaeGentianalesRubiaceae

L.Wu & Q.R.Liu, sp nov.

AB9508DD-9B19-52AC-AB50-564EF306D1BF

urn:lsid:ipni.org:names:77336995-1

Figs 1 , 2

#### Diagnosis.

The new species is most similar to *O.alatiflora*, but can be distinguished from the latter by the inflorescences which are erect from the earliest developmental stages (vs. drooping when young, then erect), the small and equally-sized calyx lobes 0.5–0.7 mm long (vs. 0.9–1.8, sometimes to 2.5 mm long and usually unequal), the longitudinal wings on the corolla tube which run to the middle of the tube and are straight (vs. wings running along entire length and obviously undulate) and the strongly reflexed corolla lobes (vs. spreading) at anthesis.

**Figure 1.**
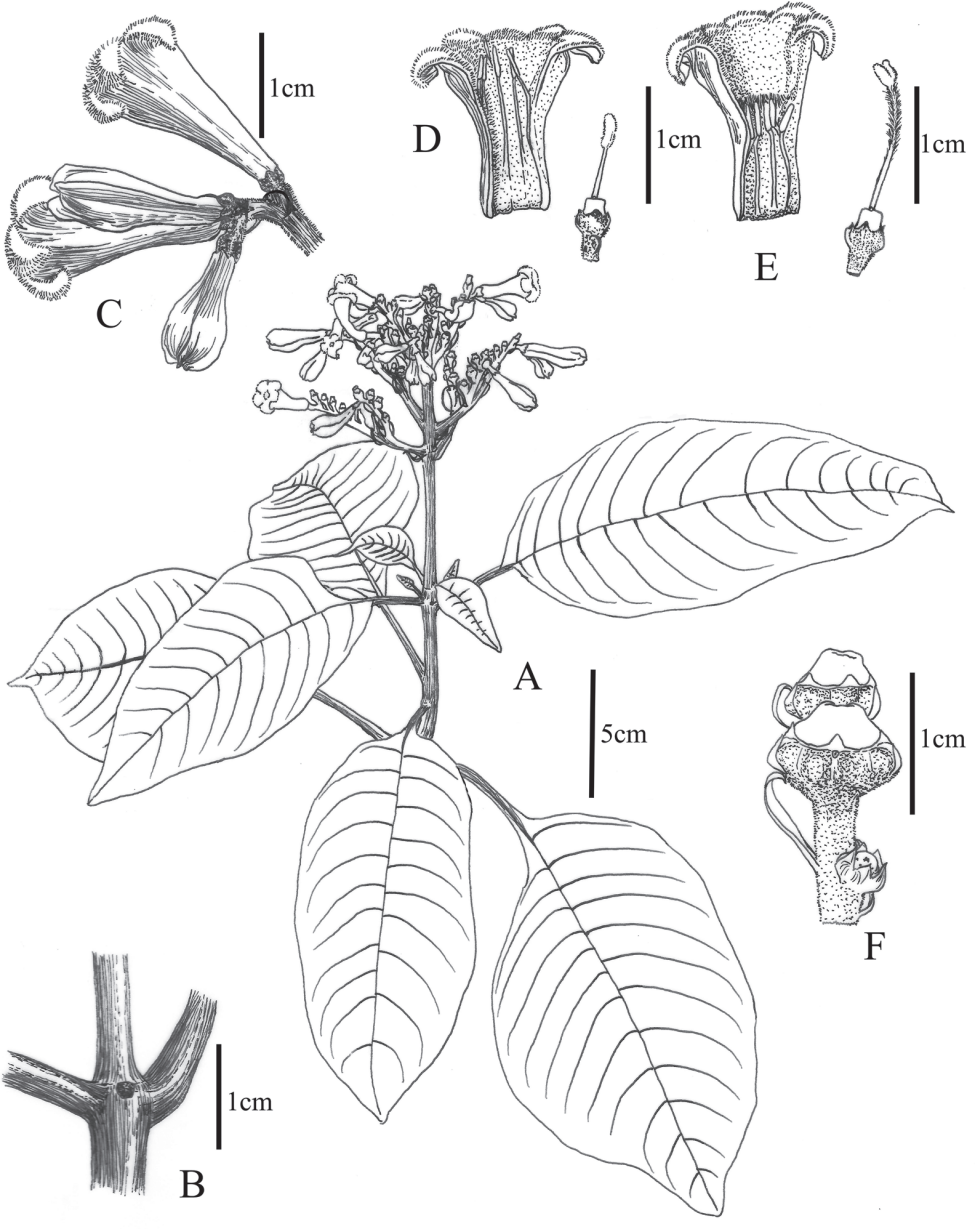
*Ophiorrhizarevoluta***A** flowering branch **B** stipule **C** part of inflorescence **D** longitudinally dissected short-styled flower **E** longitudinally dissected long-styled flower **F** capsules. Drawn from the holotype by X.Y. Zeng.

#### Type.

China. Guangxi Zhuang Autonomous Region: Napo County, Pingmeng Town, Guijiao Village, growing in limestone areas, under evergreen broad-leaved forests, rare, 23°0′30"N, 105°51′53"E, 1080 m alt., 25 Oct 2013 (fl.), *L. Wu, C. Du & S.S. Mo 4031* (holotype: CSFI 080032!; isotypes: BNU! CSFI! IBK!).

**Figure 2.**
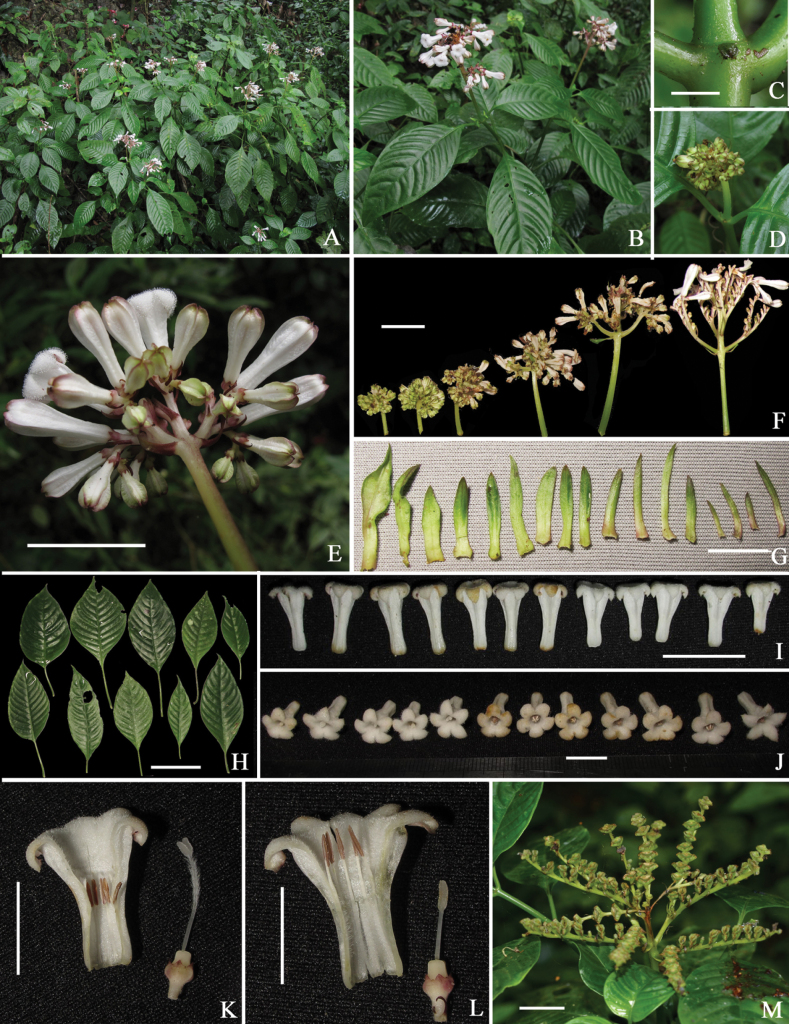
*Ophiorrhizareflexa***A, B** habit **C** stipule **D** young inflorescence **E** inflorescence in lateral view **F** inflorescences in different development stages **G** bracts from lower part to upper part of inflorescence **H** leaves **I** corollas in lateral view **J** corollas in top view **K** longitudinally dissected long-styled flower **L** longitudinally dissected short-styled flower **M** infructescence. Photos by L. Wu. Scale bars: 3 mm (**C**); 1 cm (**G, J–L**); 2 cm (**E, F, I, M**); 10 cm (**H**).

#### Description.

Perennial herbs or subshrubs, suberect, up to 100 cm tall. Stems terete to slightly compressed, glabrous. Leaves in subequal pairs; petiole 4–6 cm long, smooth; blade thickly papery, adaxially green, abaxially pale green, broadly ovate to elliptic-ovate, 11–17 × 5–8 cm, glabrous on both surfaces, base broadly cuneate to obtuse, apex acuminate or subacute, margin entire; lateral veins 9–11 on each side of the mid-rib; stipules caducous, triangular ovate, ca. 1.5 mm long, apex obtuse. Cymes terminal, erect from youngest developmental stages, many-flowered; peduncle stout, 3–6 cm long, puberulent; bracts linear-lanceolate, 8–19 × 1–3 mm, glabrous on both surfaces, apex acute; pedicels 1–3 mm long, puberulent. Flowers heterostylous. Calyx densely pilosulous to puberulent; hypanthium turbinate, 5-ribbed; lobes 5, equal, 0.5–0.7 mm long, triangular, subglabrous abaxially, with a gland in each sinus. Corolla white or sometimes slightly pink at apex, tubular-funnelform, outside glabrous; tube 1.3–1.5 cm long, outside longitudinally winged from apex to middle, wings straight, ca. 0.8 mm wide; lobes 5, ovate-triangular, ca. 4 × 3 mm, reflexed, inside densely pubescent, apex acute. Stamens 5; anthers linear, 2.5–3 mm long. Stigma bilobed; ovary 2-celled. Long-styled flowers: inside with a ring of white hairs at the middle of the corolla tube and puberulent from the middle up to the throat; stamens included, positioned near the middle of the corolla tube; style densely pubescent; stigma positioned near corolla throat, lobes ovate-elliptic, ca. 1.4 mm long. Short-styled flowers: sparsely pubescent at the middle of the corolla tube; stamens reaching slightly beyond corolla throat, not exserted; style included near the middle of the corolla tube, glabrous; stigma lobes lanceolate-elliptic, 2–3 mm long. Capsules rhomboid, ca. 4 × 9 mm, glabrous.

**Figure 3.**
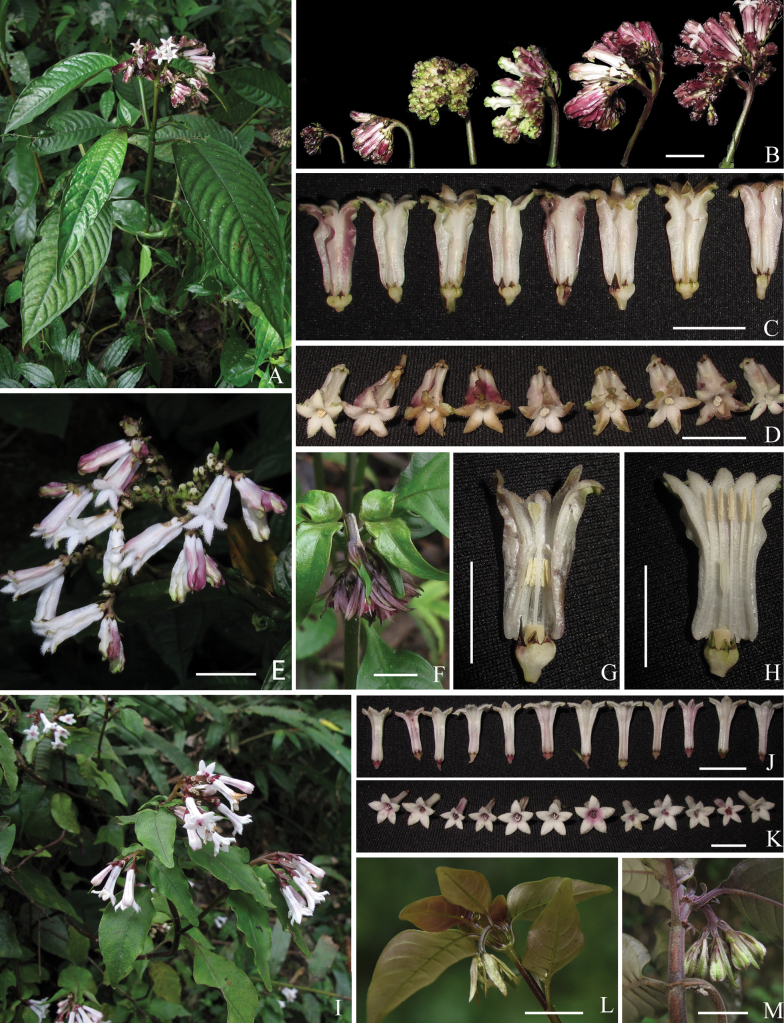
Morphological comparison of *Ophiorrhizaalatiflora* and *O.japonica***A–H***O.alatiflora***A** habit **B** inflorescences in different developmental stages **C** calyces and corollas in lateral view **D** corollas in top view **E** inflorescence **F** young inflorescence **G** longitudinally dissected long-styled flower **H** longitudinally dissected short-styled flower **I–M***O.japonica***I** habit **J** corollas in lateral view **K** corollas in top view **L, M** young inflorescence. Photos by L. Wu. Scale bars: 1 cm (**C–H, J–M**); 2 cm (**B**).

#### Phenology.

Flowering from October to January; fruiting from March to June.

#### Distribution and habitat.

**Figure 4.**
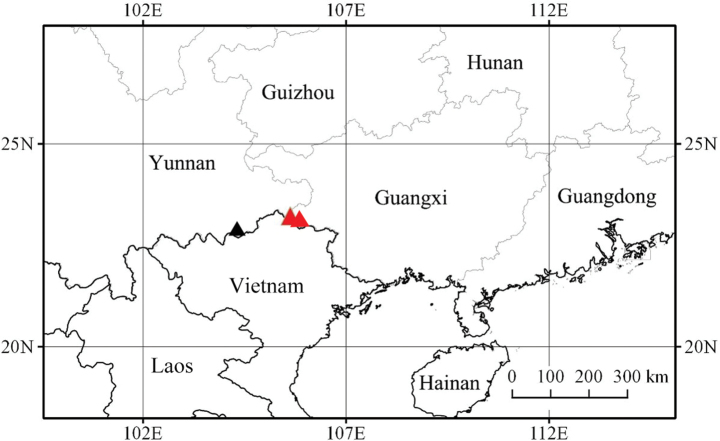
Geographical distribution of *Ophiorrhizareflexa* (red triangle, Napo County) and *O.alatiflora* (blac triangle, Malipo County).

*Ophiorrhizareflexa* grows in moist places under evergreen broad-leaved forests in the limestone region of Napo County, Guangxi, China (Fig. 4).

#### Preliminary conservation status.

Three populations of *Ophiorrhizareflexa* with more than 1000 individuals at each site have been found during our field investigations. The three sites all belong to Laohutiao Provincial Nature Reserve, which is well-protected and not under threat (Tang et al. 2013). All individuals are distributed in an area of ca. 50 km^2^ (10 × 5 km) and have remained roughly stable for the past 10 years. According to currently available data, *O.reflexa* is preliminarily assessed as Least Concern (LC) according to IUCN Categories and Criteria (IUCN 2023).

#### Additional specimens examined

**(paratypes).** China. Guangxi Zhuang Autonomous Region: Napo County, Baisheng Town, Nongming Village, 1200 m alt., 14 May 2013 (fr.), L. Wu 3706 (BNU! CSFI!); same locality as holotype, 25 Oct 2013 (fl.), L. Wu, C. Du, S.S. Mo 4033 (BNU! CSFI! IBK!); Napo County, Pingmeng Town, Guigan Village, under evergreen broad-leaved forests, 1100 m alt., 7 Jan 2014 (fl.), L. Wu 4173 (BNU! CSFI!); ibid., 10 May 2017 (fr.), L. Wu & Z.J. Wen 5891 (CSFI!).

#### Etymology.

The species epithet refers to the reflexed corolla lobes. The Chinese name is given as ‘fan-ban-she-gen-cao (反瓣蛇根草)’.

#### Notes.

*Ophiorrhiza* is a taxonomically difficult genus despite its easy distinction at genus level by the unique fruits. Misidentification or synonymy have become a major problem (Schanzer 2004; Wu et al. 2017c). As mentioned above, the reason is mainly the high morphological variation and insufficient knowledge of important characters, especially flowers (e.g. *Ophiorrhizanigricans* H.S.Lo was synonymised as *O.japonica* Blume by Duan and Lin (2007); *O.pseudonapoensis* L.Wu & Q.R.Liu has been misidentified as *O.napoensis* H.S.Lo until Liu et al. (2023)).

Based on our field investigations of *Ophiorrhiza* in China and careful studies of relevant literature and specimens, about 88% of the known species are confirmed to be distylous plants. At least 52 species have been observed by us with both long- and short-styled flowers in the same population. Meanwhile, we found that the growth pattern of the inflorescence is relatively stable in Chinese *Ophiorrhiza* species. Nearly half of the Chinese *Ophiorrhiza* species have inflorescences that are erect from the youngest developmental stages (see Fig. 2D, F), whereas the other half have inflorescences drooping when young, then gradually becoming erect (see Fig. 3B, F). In the study of *Ophiorhiza* species from the Pacific Islands, Darwin (1976) made similar observations and pointed out that the morphology of the inflorescence was taxonomically useful. However, until now, inflorescences have not received sufficient attention in most of the past studies (Lo 1990, 1999; Deb and Mondal 1997; Chen and Taylor 2011).

*Ophiorrhizareflexa* is most similar to *O.alatiflora*, both of them growing in limestone hills under dense monsoon forests. However, the former differs from the latter mainly by its erect (vs. drooping when young, then erect) inflorescences (Figs 2D, F, 3B, F), 0.5–0.7 mm long and equal calyx lobes (vs. 0.9–1.8, sometimes to 2.5 mm long and unequal, sometimes distinctly, calyx lobes; Figs 2E, K, L, 3C, G, H), strongly reflexed (vs. spreading) corolla lobes at anthesis (Figs 2E, F, K, L, 3C–E, G, H) and corolla outside with straight (vs. obviously undulate) wings from top to middle (vs. along entire length) (Figs 2E, L, 3C, G). Additionally, *Ophiorrhizareflexa* is morphologically similar to *O.japonica* Blume, the most widely distributed *Ophiorrhiza* species in China. Both of them have caducous stipules, linear-lanceolate bracts, heterostylous flowers and tubular corollas with a villous ring positioned near the middle of the corolla tube in long-styled flowers. However, the new species differs from *O.japonica* by the erect (vs. drooping when young, then erect) and lax (vs. congested or somewhat lax) inflorescences (Figs 2D, F, 3I, L, M), the strongly reflexed (vs. spreading) corolla lobes at anthesis (Figs 2E, I–F, 3I–K) and the longitudinally winged corolla with ca. 0.8 (vs. ca. 0.5) mm wide wings (Figs 2L, 3J). Further distinctive characteristics of the three species are shown in Table 1.

**Table 1.** Morphological comparison of *Ophiorrhizareflexa*, *O.alatiflora* and *O.japonica*.

	*O.reflexa*	*O.alatiflora*	*O.japonica*
Leaf blade	broadly ovate to elliptic-ovate, 11–17 × 5–8 cm, base broadly cuneate to obtuse, apex acuminate or subacute	ovate or oblong-ovate, 5–13 × 2–7 cm, base cuneate, apex shortly acuminate or subacute	ovate to narrowly lanceolate, 1–11 × 0.7–3.5 cm, base cuneate to obtuse, apex acute to acuminate
Secondary veins	9–13 pairs	7–12 pairs	4–8 pairs
Inflorescence	erect from youngest developmental stages	drooping when young, then gradually erect	drooping when young, then gradually erect
Calyx lobes	equal, 0.5–0.7 mm long	unequal, 0.9–1.8 mm long, sometimes to 2.5 mm long	equal, 0.4–1.2 mm long
Corolla	tubular-funnelform, outside longitudinally winged from top to middle, wings straight, ca. 0.8 mm wide	tubular-funnelform , outside longitudinally winged along entire length, wings undulate, 0.8–1 mm wide	tubular-funnelform to funnelform, outside longitudinal winged from top to middle, wings to 5 mm wide
Corolla lobes	ovate-triangular, reflexed, ca. 4 × 3 mm	triangular, spreading, ca. 3–3.5 × 2.5 mm	triangular to ovate, spreading, ca. 2.5–4 × 2.5–3.5 mm

## Supplementary Material

XML Treatment for Ophiorrhiza reflexa

## ﻿Additional information

### Author ORCIDs

Xiaowen Liao https://orcid.org/0000-0003-2652-2862

### Data availability

All of the data that support the findings of this study are available in the main text.

## References

[B1] Chen T Taylor CM (2011) *Ophiorrhiza*. In: Wu ZY Raven PH Hong DY (Eds) Flora of China (Vol.19). Science Press, Beijing & Missouri Botanical Garden Press, St. Louis, 258–282.

[B2] Darwin SP (1976) The Pacific species of *Ophiorrhiza* L. (Rubiaceae). Lyonia 1: 47–102.

[B3] Deb DB Mondal DC (1997) Taxonomic revision of the genus *Ophiorrhiza* L. (Rubiaceae) in Indian subcontinent. Nelumbo 39: 1–148.

[B4] Deng Y Huang Y (2012) *Ophiorrhizaloana*, a new name for *Ophiorrhizalongipes* H.S.Lo (Rubiaceae). Phytotaxa 49: 1–34. 10.11646/phytotaxa.49.1.5

[B5] Duan LD Lin Q (2007) Taxonomic notes on some species of *Ophiorrhiza* (Rubiaceae) from China. Zhiwu Fenlei Xuebao 45(6): 870–879. 10.1360/aps06098

[B6] Duan LD Lin Q (2009) Two new synonyms of *Ophiorrhizasubrubescens* Drake (Rubiaceae). Bulletin of Botanical Research 29: 3–5.

[B7] Duan LD Lin Y Lu Z (2019) *Ophiorrhizashiqianensis* (Rubiaceae), a new species from Guizhou, China. PhytoKeys 121: 43–51. 10.3897/phytokeys.121.30570 31118871 PMC6509102

[B8] Hareesh VS Sreekumar VB Kumar KMP Nirmesh TK Sreejith KA (2015) *Ophiorrhizasahyadriensis* (Rubiaceae), a new species from southern Western Ghats, Kerala, India. Phytotaxa 202(3): 219–224. 10.11646/phytotaxa.202.3.6

[B9] Hooker JD (1880) *Ophiorrhiza*. In: Hooker JD (Ed.) Flora of British India (Vol.3). Reeve & co., London, 77–84.

[B10] Hu YH Liu WJ Song XF Deng GX Nakamura K Wu L Liu QR (2021) A discussion of the relationship between *Ophiorrhizaexigua* and *O.michelloides* (Rubiaceae) with the description of a new species. Nordic Journal of Botany 39(6): e03138. 10.1111/njb.03138

[B11] Huang YS Wu L Liu Y (2017) Rubiaceae. In: Li SG (Ed.) Flora of Guangxi (Vol. 4). Guangxi Science & Technology Publishing House, 149 pp.

[B12] IUCN (2023) Guidelines for using the IUCN Red List categories and criteria, version 14. Prepared by the Standards and Petitions Committee. https://www.iucnredlist.org/resources/redlistguidelines [Accessed 28 July 2023]

[B13] Li DZ (2020) The Families and Genera of Chinese Vascular Plants (Vol. III). Science Press, Beijing, 1761 pp.

[B14] Linnaeus C (1753) Species Plantarum 1. Laurentii Salvii, Stockholm, 560 pp.

[B15] Liu Q Chen AX Liao XW Liu QR Wu L (2023) *Ophiorrhizapseudonapoensis* (Rubiaceae), a new species from Yunnan, southwestern China. Phytotaxa 607(4): 228–234. 10.11646/phytotaxa.607.4.1

[B16] Lo HS (1990) Taxonomic revision of the Chinese species of *Ophiorrhiza* (Rubiaceae). Bulletin of Botanical Research 10(2): 1–82.

[B17] Lo HS (1999) *Ophiorrhiza*. In: Lo HS (Ed.) Flora Reipublicae Popularis Sinicae (Vol.71(1)). Science Press, Beijing, 110–174.

[B18] Nakamura K Chung SW Kokubugata G Denda T Yokota M (2006) Phylogenetic systematics of the monotypic genus *Hayataella* (Rubiaceae) endemic to Taiwan. Journal of Plant Research 119(6): 657–661. 10.1007/s10265-006-0017-4 16868796

[B19] Nakamura K Denda T Kameshima O Yokota M (2007) Breakdown of distyly in a tetraploid variety of *Ophiorrhizajaponica* (Rubiaceae) and its phylogenetic analysis. Journal of Plant Research 120(4): 501–509. 10.1007/s10265-007-0089-9 17530166

[B20] Schanzer IA (2004) Systematic notes on *Ophiorrhizatrichocarpon* Blume (Rubiaceae) and some related species. Thai Forest Bulletin. Botany 32: 132–145. 10.1111/njb.03280

[B21] Schanzer IA Nabatov AA (2022) Taxonomic reassessment and lectotypification of 24 species names in *Ophiorrhiza* (Rubiaceae, Rubioideae) from Thailand. Nordic Journal of Botany 2022(2): e03280. 10.1111/njb.03280

[B22] Tang H Xue YG Wu MF (2013) Endemic and rare species of Laohutiao Nature Reserve in Guangxi, China. Journal of Guangxi Normal University: Natural Science Edition 31: 202–208.

[B23] Thiers BM (2023) [updated continuously] Index Herbariorum. https://sweetgum.nybg.org/science/ih/ [Accessed 30 July 2023]

[B24] Tu RH Li JL Wu L Hareesh VS (2018) *Ophiorrhizagaoligongensis* (Rubiaceae), a new species from southwestern China. Novon 26(4): 351–354. 10.3417/2018309

[B25] Wen ZJ Tu RH Wang LY Wu L (2019) Two newly recorded species of *Ophiorrhiza* (Rubiaceae) in China. Journal of Tropical and Subtropical Botany 27: 333–337.

[B26] Wong KM (2019) *Ophiorrhiza*. In: Middleton DJ (Ed.) Flora of Singapore (Vol.13). National Parks Board, Singapore, 215–218.

[B27] Wu L Deng YF Tan YH (2017a) Notes on *Ophiorrhizahispida* (Rubiaceae) from China. Journal of Tropical and Subtropical Botany 25: 597–600.

[B28] Wu L Hareesh VS Deng YF (2017b) Excluding *Ophiorrhizamungos* (Rubiaceae) from flora of China. Phytotaxa 2(2): 184–188. 10.11646/phytotaxa.309.2.11

[B29] Wu L Hareesh VS Yu XL (2017c) The taxonomic identity of *Ophiorrhizararior* and *O.mycetiifolia* (Rubiaceae). Phytotaxa 299(2): 261–266. 10.11646/phytotaxa.299.2.10

[B30] Wu L Tan YH Hareesh VS Liu QR (2018) *Ophiorrhizamacrocarpa* (Rubiaceae), a new viviparous species from Yunnan, south western China. Nordic Journal of Botany 36(4): 1–5. 10.1111/njb.01637

[B31] Wu L Liu WJ Nguyen SK (2019) Revision of three taxa of *Ophiorrhiza* (Rubiaceae) from China. Phytotaxa 387: 129–139. 10.11646/phytotaxa.387.2.5

